# Dr. John F. Failing

**Published:** 1906-01

**Authors:** 


DR. JOHN F. FAILING, of Los Angeles, Cal., died at his home December 6, 1905, aged 64 years. He graduated at the University of Buffalo in 1868, and practised in Grand Rapids, Mich., for some years, and then went to California on account of his health. He was hospital steward of the 128th N. Y. Infantry during the Civil War.

